# Pancreatic Lipomatosis: An Extensive Pictorial Review

**DOI:** 10.5334/jbr-btr.1014

**Published:** 2016-02-23

**Authors:** Bruno Coulier

**Affiliations:** 1 Clinique Saint-Luc, Bouge, Belgium, BE

**Keywords:** Fat, Pancreas, Fatty infiltration, Pancreatic lipomatosis, Lipomatous pseudohypertrophy, Fat replacement

## Abstract

Pancreatic lipomatosis – also commonly called fat replacement – represents the most frequent benign pathologic condition of the adult pancreas. Most cases remain asymptomatic, and only some rare extreme degrees of lipomatosis or fat replacement may lead to exocrine pancreatic insufficiency.

The precise etiology of the entity remains unclear, and the condition has been found associated with several diseases comprising diabetes mellitus, metabolic syndrome, acquired or hereditary pancreatitis, alcoholic hepatitis, cystic fibrosis and condition comprising increasing age, body mass index, or more precisely visceral fat index, and use of steroid therapy. Numerous cases are also associated with condition compromising the permeability of the pancreas ductal system, such as intraductal calculus, pancreatic tumors, and congenital or experimental stenosis.

Uneven lipomatosis and fat replacement are also common presentations and responsible for the great diversity of imaging features. The reasons for uneven presentations are controversial and probably interweave embryologic or ductal hypotheses. Lipomatous pseudohypertrophy of the pancreas represents the most extreme situation of pancreatic lipomatosis and is considered, probably in a contestable way, as a rare, specific, and distinct entity.

We hereby present an extensive pictorial review of the broad spectrum of imaging features of this entity. The images are extracted from a compilation of cases collected in our department over more than a decade. We review and discuss the embryologic and ductal hypothesis, the differential diagnosis. Finally, we illustrate some unusual presentations and evolutions.

## Definitions and Background

The pancreas is both an endocrine and an exocrine gland. The exocrine component accounts for about 80 per cent of the total glandular volume and is mostly constituted of two different types of cells: acinar cells (primarily secreting digestive enzymes) and ductal cells (mainly secreting fluids and electrolytes). The endocrine component comprises the typical islets of Langerhans, which contain several types of cells scattered within the exocrine tissue [1].

*Lipomatosis* and *fat replacement* of the pancreas are the most frequent benign pathologic conditions of the adult pancreas [1,2,3]. Classically, the phenomenon causes an increasing hypodensity of the pancreas on CT and a typical hyperechogenicity on ultrasound (US) examination.

Accumulation of fat within the pancreas (lipomatosis) and replacement of various portions of the pancreatic gland by fat (fat replacement) have been named with various synonyms: pancreatic lipomatosis, fatty replacement, fatty infiltration, fatty pancreas, lipomatous pseudohypertrophy, non-alcoholic fatty pancreatic disease, and pancreatic steatosis [4]. These synonyms are sources of confusion.

On the basis of the various imaging findings, one could be inclined to use the term of “lipomatous infiltration” when the pancreatic glandular islands appear dissociated by fatty tissue or when the density (CT), echogenicity (ultrasound), or signal (MRI) are diffusely modified. When the pancreatic islands seem to have disappeared or been grossly replaced by fat, one might be more prone to prefer the vocable “fat replacement”.

Similarly, one may also be inclined to use the term “lipomatous infiltration” when the process seems reversible and reserve the vocable of “fat replacement” to cases showing a likely irreversible disappearance of glandular islands [4,5].

Nevertheless, there is a massive lack of significant histopathological studies to prove or disprove that lipomatosis and fat replacement of the pancreas are (i) distinctive entities, (ii) associated entities in variable proportions in a definite case, or (iii) expressions of the same disease. Therefore, to simplify the lecture of this pictorial review, the terms *fat replacement* and *lipomatosis* of the pancreas have been replaced by the simplified term *fatty infiltration*, or “FI”, throughout. It highlights that FI of the pancreas may be uniform, unevenly distributed, or confined to one region of the pancreas (focal FI) [1,6].

## Clinical Findings

Most of limited pancreatic fat deposits have no major clinical significance. On the contrary, extreme degrees of FI of the pancreas may be associated with a significant depression of pancreatic function which may secondarily lead to exocrine pancreatic insufficiency. This situation associates maldigestion of nutrients and clinical symptoms comprising chronic diarrhea, steatorrhea, and weight loss without abdominal pain or diabetes [1].

Nevertheless, only a few case reports have suggested a direct relation between pancreatic FI and exocrine pancreatic insufficiency [7], and a proper demonstration of this association is yet to be established. Further functional studies are necessary to establish the exact degree of FI capable of causing symptomatic exocrine insufficiency. For evidence, most of the cases reported in this pictorial review were only fortuitously diagnosed. Symptoms of pancreatic exocrine insufficiency were commonly absent or at least not diagnosed because of being clinically occult. Only a proportion of patients had the ascertainment of a low blood level of lipase in laboratory tests.

## Etiology

**Figure 1:**
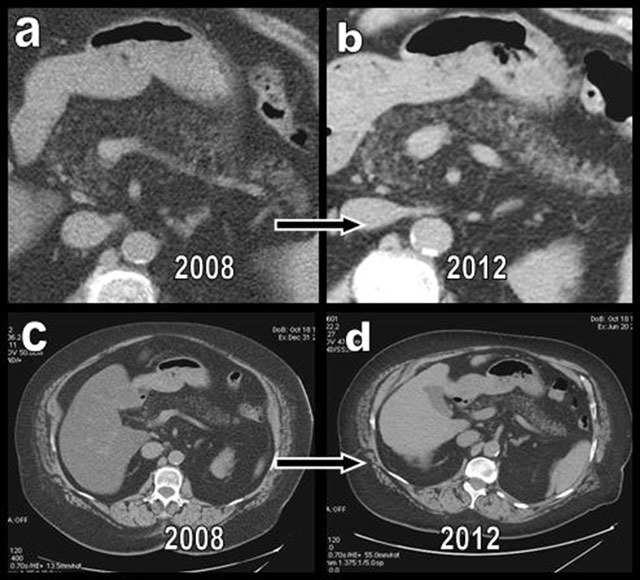
Major diffuse FI (type 2b) was found in a 75-year-old women presenting with morbid obesity and chronic diabetes mellitus (a and c). A significant regression of the global FI was found after a 4-year period (b and d). Both the dissociation of the pancreatic lobules by fat and the global volume of the organ had regressed, and the patient had lost more than 25 kg. Hepatic steatosis had also drastically regressed.

FI is a benign disease that has no single etiology [1,2,7,8,9]. The entity has been associated with many diseases and conditions. Age and obesity correlate significantly to the grade of FI [9,10]. As a consequence, FI usually directly correlates with the patient’s body mass index (BMI). More precisely, the correlation is better between FI and the *visceral fat index*, which is nevertheless more complex to evaluate than the BMI or weight of the patient. In other terms, the amount of visceral fat tissue is a better indicator and predictor of FI of the pancreas than the BMI alone (Figure 1) [1].

FI also correlates with the presence of diabetes mellitus or acquired or hereditary pancreatitis [3]. Although FI is associated with the presence of diabetes mellitus; it is not specifically due to the disease itself. The typical islets of Langerhans are paradoxically noteworthy for being resistant to FI.

FI is highly associated with the metabolic syndrome [4], an emerging syndrome consisting of the association of at least three of following features: hyperinsulinemia; hypertension; hypercholesterolemia; obesity; and hyperglycemia. FI of the pancreas has also been observed in patients presenting with alcoholic hepatitis or under steroid therapy [1].

Major FI of the pancreas is also the most frequent CT pattern in adolescents and adults with cystic fibrosis [11,12,13].

**Figure 2:**
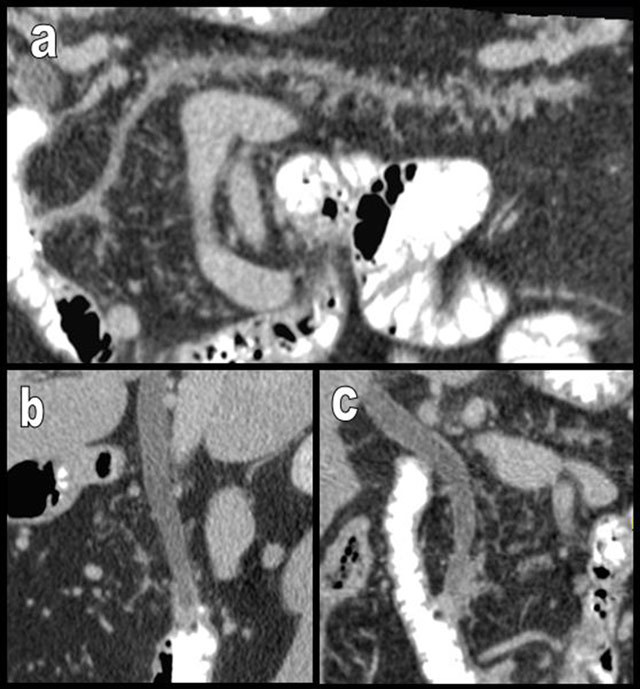
Curvilinear (a), sagittal cephalic (b), and coronal (c) reformatted views (MPR) in a case of complete FI of the pancreas (type 2b). Pseudohypertrophy is not associated. The patient, a 50-year-old man, had only a mild biologic pancreatic exocrine insufficiency. Only a small number of glandular acini persist along the main and secondary ductal network. The patient had previously undergone surgical resection of a duodenal neuroendocrine tumor situated partially on the papilla, and massive FI was already seen before surgery (not illustrated). The hypothesis of a chronic obstacle on the flow of bile duct or main pancreatic duct as the main causal factor (“ductal hypothesis”) of the massive diffuse FI is supported by this case.

**Figure 3:**
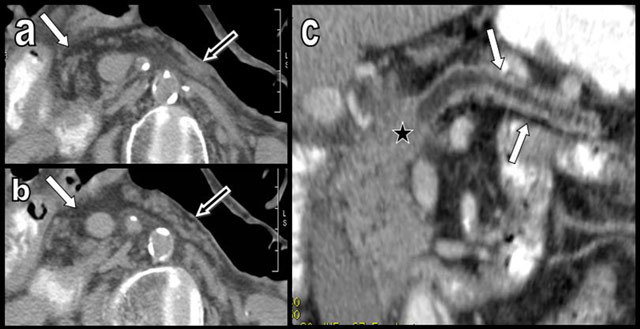
Aging may cause FI (white arrows on a and b) but also atrophy of the pancreas (black arrows), as illustrated in this 100-year-old woman. Major atrophy of the pancreatic corpus or tail (white arrows on c) is more common than lipomatosis in the case of chronic obstruction of the Wirsung by a tumor, as illustrated in this 88-year-old man presenting with a small corporeal adenocarcinoma (black star). In the case of true pancreatic atrophy, the surrounding organs converge to occupy the pancreatic bed (white arrows on c).

**Figure 4:**
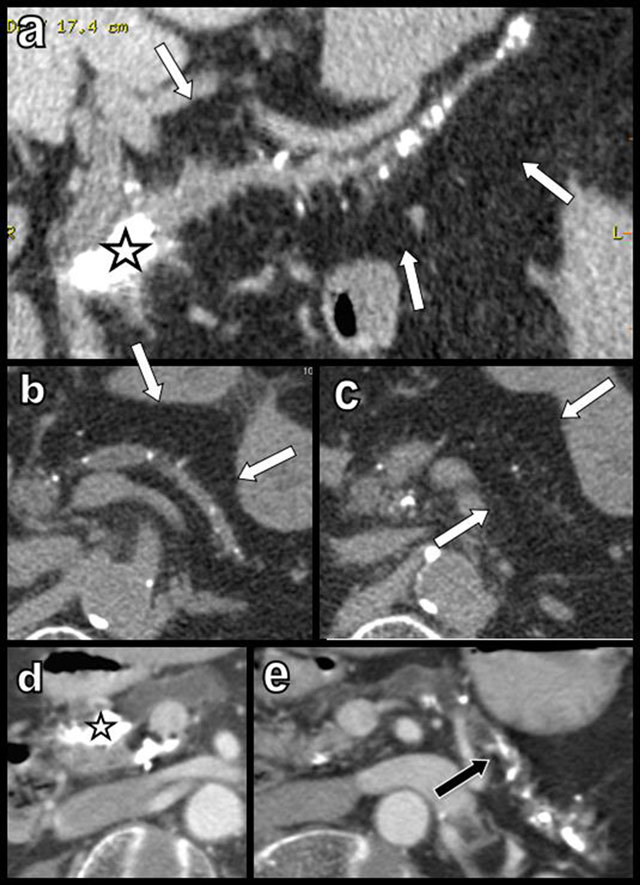
Global and massive FI (type 2b) are found in a 60-year-old woman, resulting from calcifying chronic pancreatitis with chronic obstruction of the main duct by a large calcification (star). Curvilinear reconstruction (a) and axial views (b and c) are shown. The primitive pancreatic bed (or volume) is preserved (white arrows). An extremely similar case of calcifying chronic pancreatitis (d and e) has evolved to massive atrophy of the pancreas (black arrow) instead of FI. The reason for this difference of evolution is unknown.

Numerous cases of FI have been found associated with pathologies related to or affected by permeability of the pancreas ductal system. So marked FI, with or without glandular atrophy, has commonly been observed in adult patients, secondary to pancreatic duct obstruction by intraductal calculi or pancreatic tumors (Figures 2, 3 and 4) [7]. Congenital stenosis of the pancreatic duct also can result in FI of the parenchyma in many cases [14]. Previous animal experimentations have already demonstrated that the ligation of the pancreatic duct could result in atrophy and lysis of the pancreatic acini, whereas islets of Langerhans were relatively preserved. The ductal cells slowly decreased in number and became rare due to cell death. However, the pancreas became gradually enlarged by intralobular fatty replacement until 16 weeks [15].

## Uneven Fatty Infiltration

Uneven FI of the pancreas is a very common – and in reality the most common – presentation of FI, and many cases have been reported [2,6,7,16]. Although uneven FI of the pancreas may affect any portion of the gland, it is usually more frequent and prominent in the anterior pancreatic head (APH). The posterior pancreatic head (PPH) of pancreas and the uncinate process (UP) are usually spared or at least more refractory to FI [2,7]. The most refractory area to FI is a very small focal area around the common bile duct [16].

**Figure 5:**
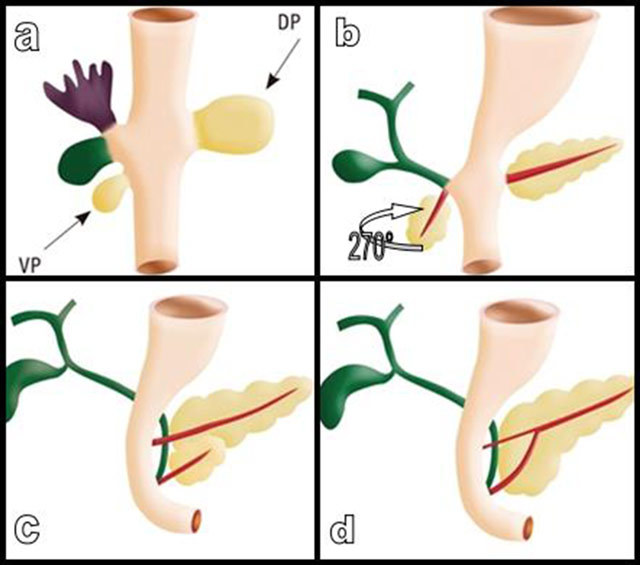
The pancreas develops from the fusion of the dorsal (DP) and ventral (VP) pancreatic buds (a). The VP is common with bile duct (in green). At six weeks of gestation, the VP undergoes a clockwise rotation of 270° (b) to finish at the posteroinferior side of the DP (c). It thus becomes anatomically posterior. The DP gives rise to the APH of the pancreas, in addition to the body and tail, while the rotating VP develops into the PPH and UP. The fusion of the pancreatic buds is accompanied by anastomosis of the ducts (d). The main duct of the VP fuses with the main duct of the DP and becomes the dominant and more constant Wirsung’s duct. Thus, the Wirsung’s duct drains portions of the pancreas of different embryologic origin. The distal part of the main DP duct partially regresses to form the accessory duct of Santorini, which opens into the minor duodenal papilla.

**Figure 6:**
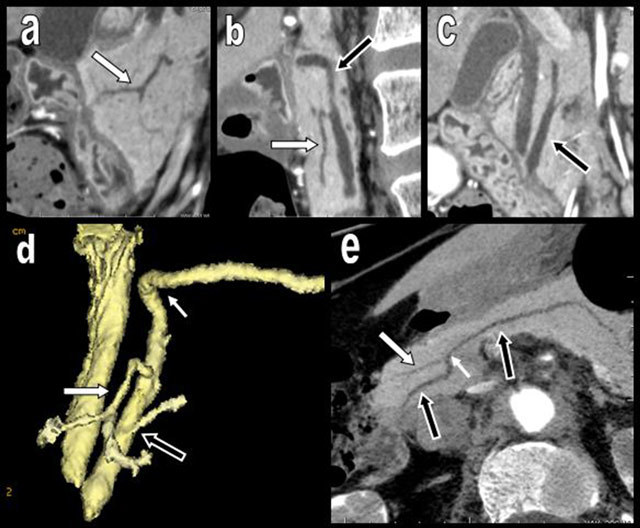
Ductal anatomy of the pancreas. During development, the canal of the corporeal caudal pancreas (dorsal bud) fused with the canal of the PPH (b) (ventral bud) to form the main canal of Wirsung (black arrow). This fusion expIains its typical bayonet shape (small white arrow). The duct of the ventral head (a) (originating from the distal dorsal bud) becomes the accessory canal of Santorini (white arrow). The classical spatial arrangement of these ducts is well illustrated on sagittal reconstruction of the head (b), 3-D volume rendering view (d), and thick minimal intensity projection (MIN) of the pancreas (c).

This anatomic distinction between the APH and the PPH and UP is of primordial importance in terms of embryology. The human pancreas develops from the fusion of the dorsal and ventral pancreatic buds (Figure 5). The ventral bud is common with the bile duct. At six weeks of gestation, it undergoes a clockwise rotation of 270° to finish at the posterior inferior side of the dorsal bud. Thus, it becomes anatomically posterior [17,18]. The dorsal pancreatic bud gives rise to the anterior part of the head (APH) of the pancreas, in addition to the body and tail, while the rotating ventral pancreatic bud develops into the posterior part of the head (PPH) and uncinate process (UP). This fusion of pancreatic buds is associated with anastomosis of their respective ducts (Figures 5 and 6) except in about 4–10 per cent of patients that will present with pancreas divisum. The main duct of the ventral bud enters in preponderant communication with the main duct of the dorsal bud. The point of fusion produces between the isthmus and the head and results in the creation of the dominant and more constant Wirsung’s duct and explains its bayonet appearance [19] (Figure 6). The Wirsung’s duct consequently drains portions of the pancreas of different embryologic origin: the PPH (originating from the ventral bud) and the corpus and tail (originating from the dorsal bud). The proximal part of the main dorsal pancreatic duct partially regresses to form the accessory pancreatic duct, or Santorini’s duct, which opens into the minor duodenal papilla.

**Figure 7:**
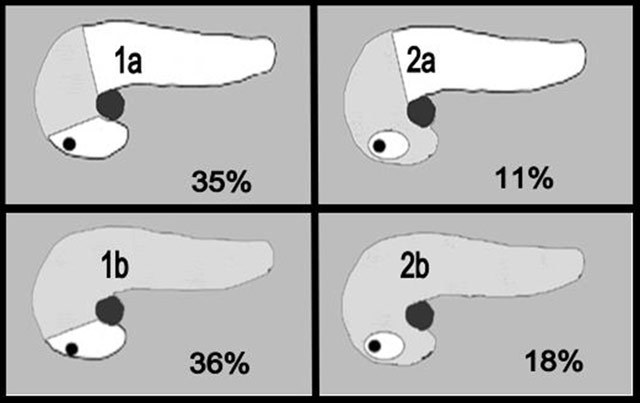
Schematic classification of FI proposed by Matsumoto. FI is figured by grey areas. In type 1 (71% of cases), the PPH and the UP are spared from FI. In type 1a (35% of cases), FI is limited to the APH. And in type 1b (36% of cases), FI of the APH is associated with FI of the body and tail. In type 2 (29% of cases), FI involves all the head except a very small refractory focal area around the common bile duct. In type 2a (11% of cases), FI is limited to the entire head. And in type 2b (18% of cases), FI of the head is associated with FI of the body and tail. Thus, FI type 2b represents FI of nearly the entire pancreas (except a very small focal area around the common bile duct).

Uneven FI of the pancreas was classified by Matsumoto in 1995 (Figure 7) into two types, and each of these was secondarily classified into two subgroups [16]. In type 1 (71% of cases), the PPH and UP are spared from FI. In type 1a (35% of cases), FI is limited to the APH. And in type 1b (36% of cases), FI of the APH is associated with FI of the body and tail.

In type 2 (29% of cases), FI involves all the head except a very small refractory focal area around the common bile duct. In type 2a (11% of cases), FI is limited to the entire head. And in type 2b (18% of cases), FI of the head is associated with FI of the body and tail. Thus, type 2b represents FI of nearly the entire pancreas (except a very small focal area around the common bile duct). Uneven FI is thus found in approximately 82 per cent of FI cases (types 1a, 1b, and 2a).

Uneven FI of the two different embryologic portions (APH and PPH) of cephalic head (types 1a and 2a) is thus found in 46 per cent of cases of uneven FI of the pancreas. The reason for this difference in FI capabilities of the two superposed and embryologically distinct portions of the head of the pancreas remains unclear but may be partially explained by their constitutional histological differences in relation to their different embryologic origin [6]. It has been evidenced that the embryologic ventral pancreas that becomes the PPH and UP has smaller, densely packed acini, smaller exocrine cells, scanty or absent intraparenchymatous fat cells, and more interlobular fibrous tissue than the embryologic dorsal pancreas [6,16].

It is our opinion that this “histologic embryologic hypothesis” cannot alone explain the frequent uneven FI of the pancreas. Indeed, this hypothesis does not take into account that the pancreatic body and tail, which are frequently respected by FI in cases of isolated APH FI (type 1a) or global cephalic FI (type 1b) – in fact, in 71 per cent of cases when types 1a and 1b are cumulated – have the same embryologic origin. Thus, another hypothesis than the embryologic hypothesis must be found for these cases.

After fusion of the two embryologic buds of the pancreas, the proximal part of the main dorsal pancreal duct partially regresses to form the accessory pancreatic duct of Santorini which opens into the minor duodenal papilla [19]. This minor papilla is substantially smaller than the major papilla and may then be subjected to a possible overload of secretory capacity [19]. Studies have recently found a significant correlation between patency of the accessory pancreatic duct and acute pancreatitis, the patency of the accessory duct being significantly lower in patients with pancreatitis (17%) than in control cases (43%) [19]. Patency of the accessory duct is thus extremely variable and appears reduced or absent in many patients.

Moreover, in patients presenting with a pancreas divisum (4% to 14 % of the population), most of the main dorsal duct remains separated from the ventral pancreas and consequently drains exclusively through the minor papilla [18]. It is thought that the disproportion between the small caliber of the minor papilla and the large amount of secretions from the dorsal part of the gland may lead to a relative outflow obstruction from the dorsal pancreas, leading to pain or pancreatitis. Controversies exist, but positive correlations between pancreas divisum and pancreatitis have been shown [18,19,20].

The accessory duct of Santorini drains all the APH, which is precisely the portion of the gland that is more prone to FI. The frequent preponderance of isolated massive lipomatosis of the APH has fuelled the hypothesis that FI is also probably caused by the poor quality of the drainage by the accessory canal of Santorini. This “ductal hypothesis” probably coexists and interferes with the “embryologic hypothesis” in cases of uneven lipomatosis.

### Imaging of Fatty Infiltration of the Pancreas

**Figure 8:**
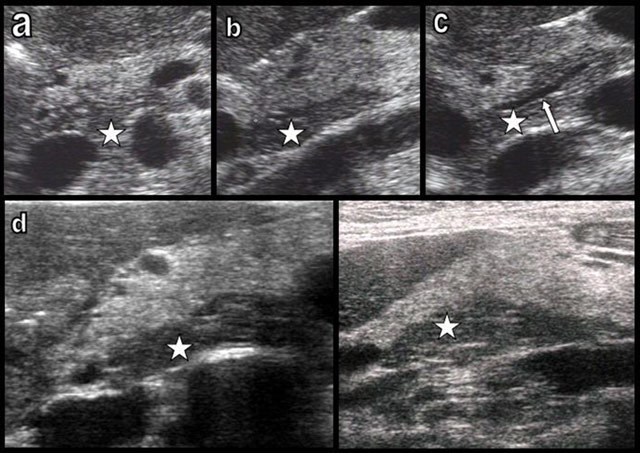
Uneven FI of the cephalic pancreas (type 1a) is commonly found during abdominal ultrasound, especially in middle-aged women. Classical transverse views (a, c, and d) and sagittal views (b and e) are shown. The biliary duct and the distal Wirsung (white arrow) classically run in the PPH (embryological ventral bud), which more commonly appears hypoechoic (star) because it is lesser affected by FI.

Uneven FI of the cephalic pancreas is commonly found not only on advanced imaging techniques but also on pancreatic US [17,20]. The ventral embryologic pancreas which tends to remain free from FI frequently appears more hypoechoic than the dorsal part (Figure 8). During US, it is primordial to be continuously aware of this classical pattern because many pathologic pancreatic processes are also frequently spontaneously hypoechoic, including carcinoma, metastases, lymphoma, and carcinoid tumors as well as focal and segmental acute and chronic pancreatitis [17].

The reason for the higher sensitivity of US to detect uneven cephalic FI is considered to be in relation to the generally higher sensibility of US to detect subtle differences in fat in various tissues. It is also a common observation in the liver in which US more easily detects the hyperechoic area of circumscribed steatosis and hypoechoic area of tissue respected by steatosis than CT [21].

During US, FI of the pancreas appears hyperechoic – and not hypoechoic as classically observed in lipoma. The reason is that it is not fat itself that determines the echogenicity but the architectural modification due to the development of adipocytes within the interlobular septa. It is the alternation of glandular and fatty interfaces that is responsible of hyperechogenicity [17].

The echogenicity of the pancreas gradually increases with age and obesity. Uneven FI of the pancreatic head is rarely before the age of 25 years and is most frequently found in middle-aged females presenting with a moderately echoic pancreas [17]. When the pancreas becomes very lipomatous and thus extremely hyperechoic, the performance of US to diagnose uneven FI of the cephalic head nevertheless drastically reduces because of a phenomenon of saturation but also because the transmission of US through the hyperechoic anterior head drastically reduces. The same situation is found in cases of massive liver steatosis.

On the contrary, the more the pancreas is infiltrated or replaced by fat, the more CT can easily diagnose the entity. CT thus becomes the imaging modality of choice in massive FI of the pancreas.

**Figure 9:**
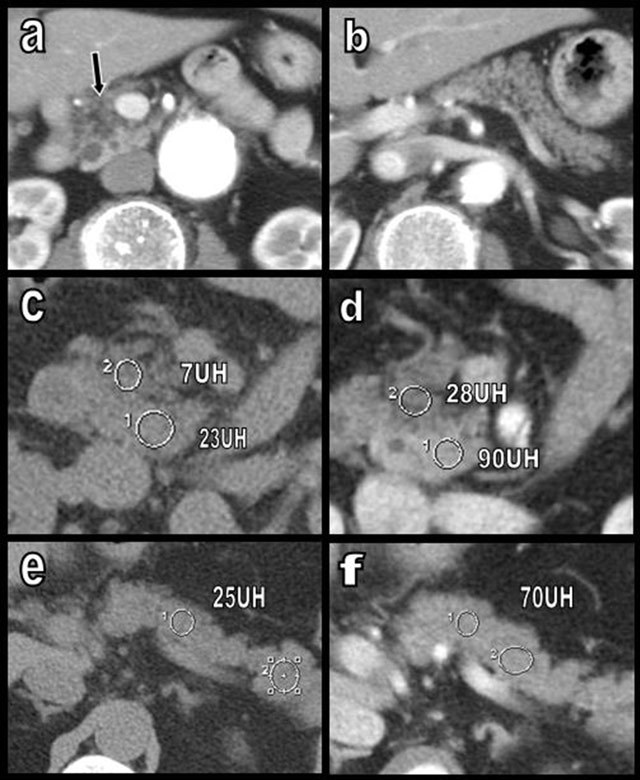
Typical case of uneven FI (a and b) limited to the APH (type 1a) (black arrow). However, the typical “negative” density of fat is not so obvious because the measures of density invariably include residual glandular acini. These acini also enhance with intravenous contrast of the rest of the gland. Nevertheless, the global contrast enhancement of the lipomatous area (from 7 UH (c) to 28 UH (d)) remains inferior to that of the rest of the gland (from 23–25 UH (e) to 70–90 UH (f)). This situation may mimic a pancreatic cancer, especially when the signal-to-noise ratio of the image is poor or if the slice thickness is not appropriated. In difficult or ambiguous cases, ultrasound or MRI can help in the differential diagnosis.

In clinical practice, pancreatic FI is generally unambiguously diagnosed through CT when the FI is sufficiently sharp to show typical negative attenuation Hounsfield values. Nevertheless, if the degree of focal FI remains mild, the attenuation may be insufficient and may simulate a cystic or a hypodense tumoral process [2,7]. Moreover, on post-contrast images, the normal pancreatic parenchyma entrapped between FI areas may show significant contrast enhancement (Figure 9), which may simulate a tumoral mass [2,7].

Classically, the different patterns of uneven FI described by Matsumoto are commonly found during clinical CT imaging:

The PPH and UP are spared from FI in type 1

**Figure 10:**
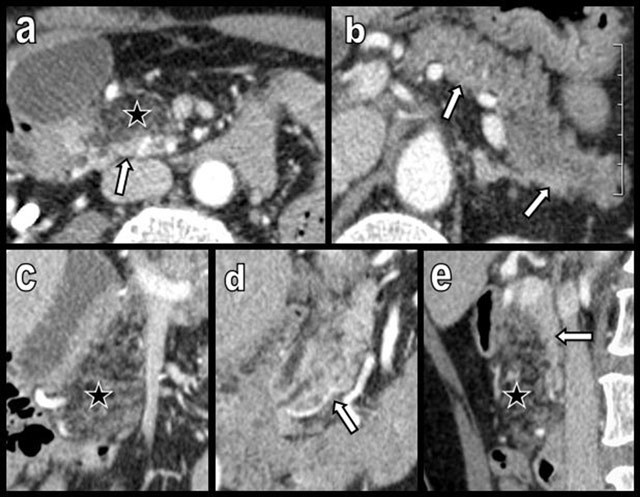
Figures a–e illustrate axial (a and b), coronal (c and d), and sagittal (e) pancreatic views of a 70-year-old woman with morbid obesity and type 2 diabetes mellitus. FI only electively involves the APH (black star) (type 1a). The hypodensity of the lipomatous APH drastically contrasts with the normal density of PPH, UP, and corporeal caudal pancreas (white arrows).

**Figure 11:**
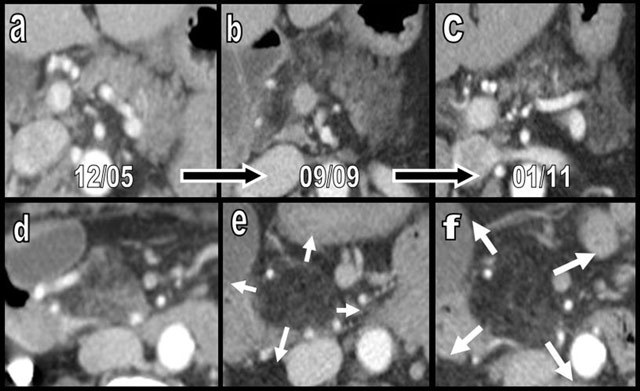
Rapid progression of diffuse FI, predominantly affecting the APH (type 1a) in a 70-year-old woman. The patient was imaged 3 times during a 6-year period because of recurrent episodes of ischemic colitis. On axial slices, FI progressively increases in all portions of the tissue (a–c) but drastically predominates on the APH (c–e), where it is associated with progressive cephalic enlargement and focal “pseudohypertrophy” (white arrows).

Type 1a (35% of cases) in which FI is limited to the APH is illustrated in Figures 10 and 11.

**Figure 12:**
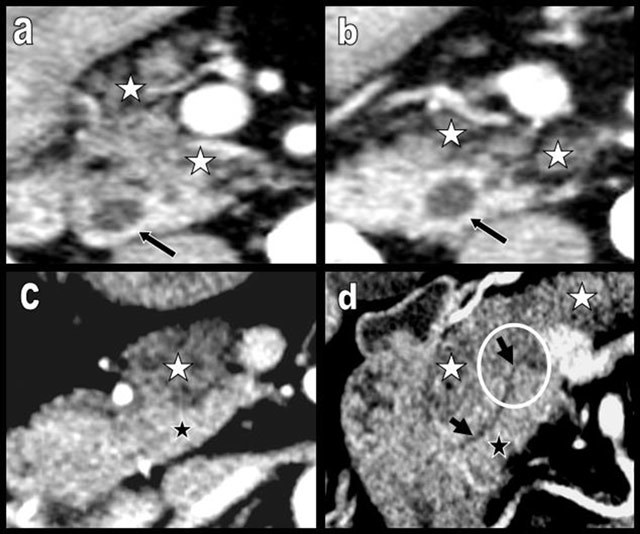
Typical aspects of the pancreatic head in FI type 2b (a and b). Both the APH and a significant proportion of the PPH and UP are infiltrated by fat. Only a very small restricted ovoid area of pancreatic tissue around the biliary duct remains unaffected (black arrow). A subtle but typical case of FI type 1b (figures c and d). The APH and corporeal caudal portions of the gland (white stars) appear more hypodense because of lipomatous. They contrast with the higher density of the PPH (black star). The abrupt change of density is found just at the level of the cephalo-isthmic junction (white circle), although these two areas are drained by the same normal canal of Wirsung (black arrows). This case supports an embryologic histologic explanation for the uneven lipomatosis.

Type 1b (36% of cases) in which FI of the APH is associated with FI of the body and tail is illustrated in Figure 12.

FI involves all the head except a very small refractory focal area around the common bile duct in type 2.

Type 2a (11% of cases) in which FI is limited to the entire head is the rarest type.

**Figure 13:**
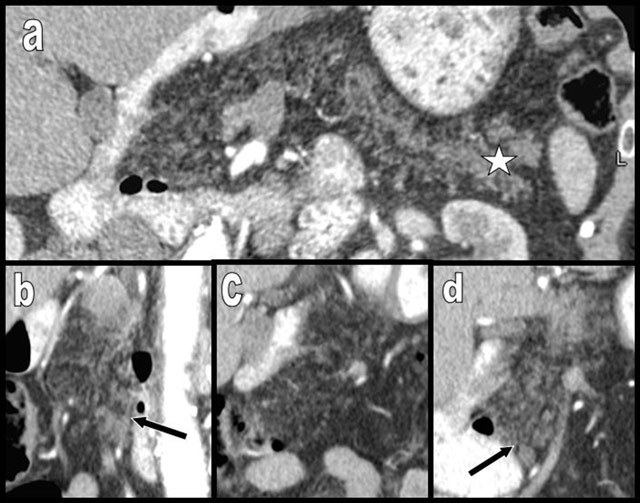
Curvilinear reconstruction (a), sagittal cephalic (b), and anterior (c) and posterior (d) coronal cephalic views of a nearly compIete extensive and homogenous FI (type 2b) found in a rather young patient (60 years) with a normal BMI, no diabetes, and no clinical or biological signs of pancreatic exocrine insufficiency. Discrete enlargement of the organ is also present. FI is rather homogenous except for a discretely lesser involution of the posterior head (black arrows) and the end of the tail (star).

**Figure 14:**
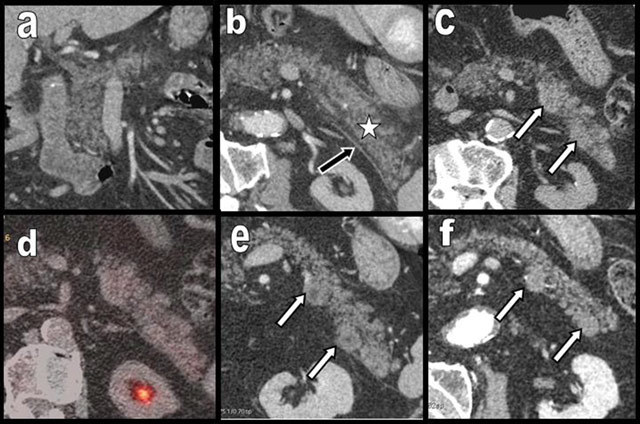
Coronal cephalic (a) and axial oblique caudal views (b) show subtle signs of acute caudal pancreatitis superimposing on diffuse FI of a 75-year-old patient (type 2b) suffering from colonic carcinoma. Discrete edema is found between the glandular lobules (white star), and a small linear effusion (black arrow) is found in the anterior pararenal fascia. Follow-up studies – unenhanced CT at day 5 (b) and contrast enhanced CT at day 30 (e) – show inflammatory agglutination of lobules mimicking pseudotumoral nodules (white arrows). Fortunately intercurrent 18F-FDG PET/CT, performed in the follow-up of colonic carcinoma at day 15 showed negative FDG uptake (d). Slow regression of the necrotizing pseudotumoral nodules is clearly visible after 10 months (f).

Type 2b (18% of cases) represents FI of nearly the entire pancreas (except a very small focal area around the common bile duct) (Figures 1, 2, 4, and 12,13,14).

**Figure 15:**
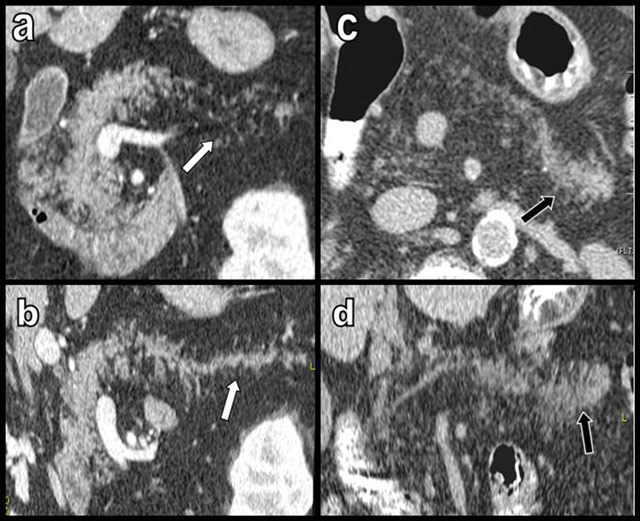
Atypical and unclassifiable cases of uneven FI. Coronal oblique (a) and curvilinear (b) reformations in an 81-year-old patient presenting with massive FI of the tail only (white arrow). Axial oblique (c) and curvilinear (d) reformations in other 88-year-old patient presenting with massive FI of the head and corpus contrasting with a relative respect of the tail (black arrow).

**Figure 16:**
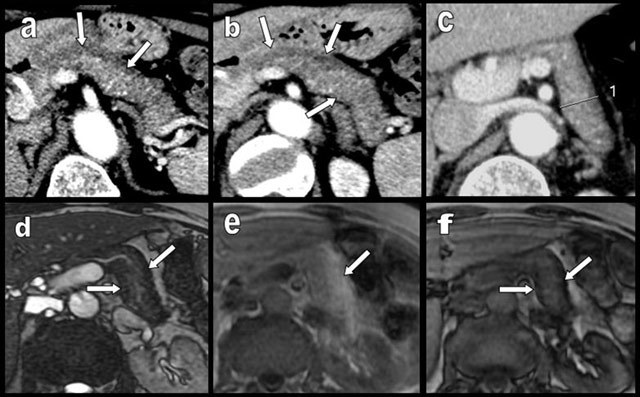
Atypical peripheral uneven FI. The gland (a and b) is surrounded by an hypodense band mimicking peripancreatic edema or a subtle effusion on contrast enhanced CT views (white arrows on a and b). This hypodense band was absent 2 years before, and the pancreatic tail was thinner (c). The peripheral band is hypointense on a T2-weighted image (d), thus excluding edematous effusion (white arrows on d). Axial in-phase images (e) show normal signal intensity in the body of the pancreas, higher signal in the peripheral gland, markedly on the anterior side (white arrow on e), with a drop of signal intensity on opposed-phase imaging (white arrows on f) revealing atypical peripheral uneven pericaudal FI.

**Figure 17:**
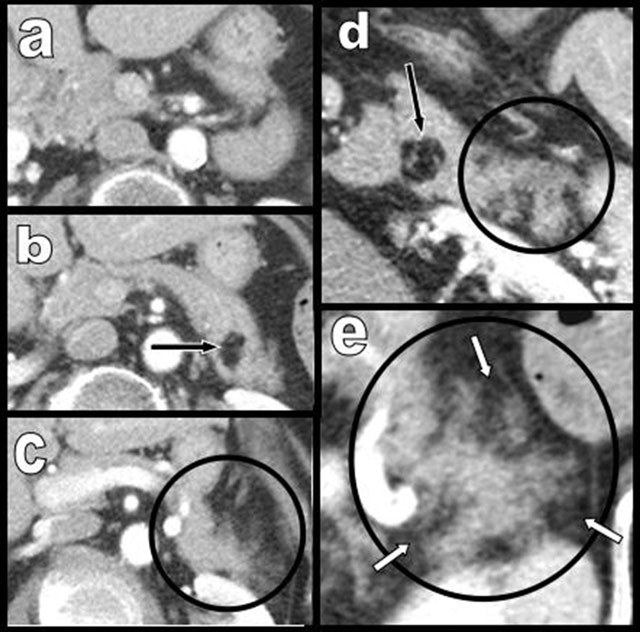
Figures a–e illustrate an unusual case of FI exclusively affecting the distal tail of the pancreas (black circle). This atypical FI is associated with elective pseudohypertrophy. An isolated small lipoma of the tail is associated (black arrows on b and d). The rest of the gland appears normal. This rare type of caudal FI was very stable over a 5-year period.

As illustrated in this pictorial review, other atypical and unclassified presentations of uneven FI may also be found (Figures 15,16,17).

**Figure 18:**
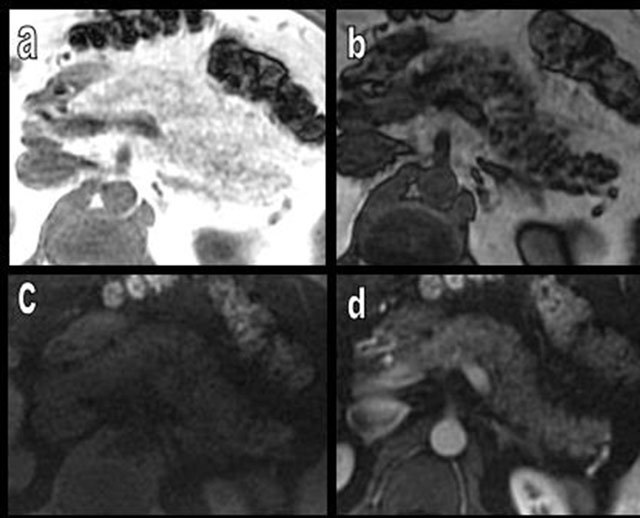
MRI of the pancreas performed to exclude choledocholithiasis in a patient who has suffered from acute pancreatitis two months before. Transverse in- and out-phase MR images respectively show a typical global hyper- (a) and hypointensity (b) of the pancreas. A T1-weighted image with fat suppression (c) confirms hypointensity of the pancreas due to massive FI. A T1-weighted image with fat suppression after gadolinium enhancing (d) shows homogenous enhancement of the entire pancreas.

**Figure 19:**
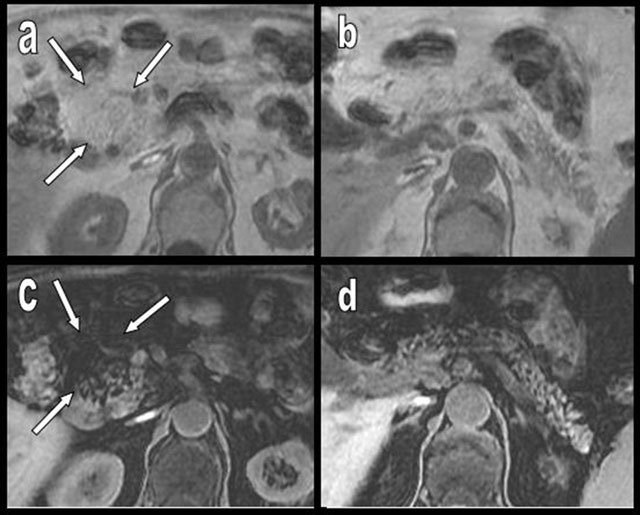
Typical MRI aspects in a case of diffuse FI of the pancreas. FI predominates on the APH (type 1a) with glandular enlargement (white arrows). On T1-weighted images (a and b), the lipomatous APH has a typical high signal. Drastic extinction of the signal is found on T1-weighted images with fat suppression (c and d).

In atypical or ambiguous cases, chemical shift MRI may be helpful to make the differential diagnosis [2,7]. Chemical shift MRI has an advantage over CT in confirming the presence of focal FI of the pancreas. A characteristic loss of signal intensity on opposed-phase T1-weighted gradient-echo image as compared with corresponding in-phase image confirms the presence of microscopic lipid within the focal pancreatic mass [7]. This histologic condition is, in general, not present in pancreatic cancer [2]. MRI sequences with fat suppression are also useful for the diagnosis of FI (Figures 16, 18, and 19).

Due to the benignity and rarity of severe FI of the pancreas, the findings during endoscopic retrograde cholangiopancreatography (ERCP) are not well documented. In minimal FI, the pancreatogram is normal [9]. In severe FI, ERCP may show stenosis or abrupt obstruction at the body and tail portions. The stenotic pancreatic main duct is typically smooth and has an elongated form. Obstruction has to be distinguished from pathologic obstruction due to agenesis, chronic pancreatitis, or cancer. Today these rare findings could be preferentially described on magnetic resonance cholangiopancreatography (MRCP).

**Figure 20:**
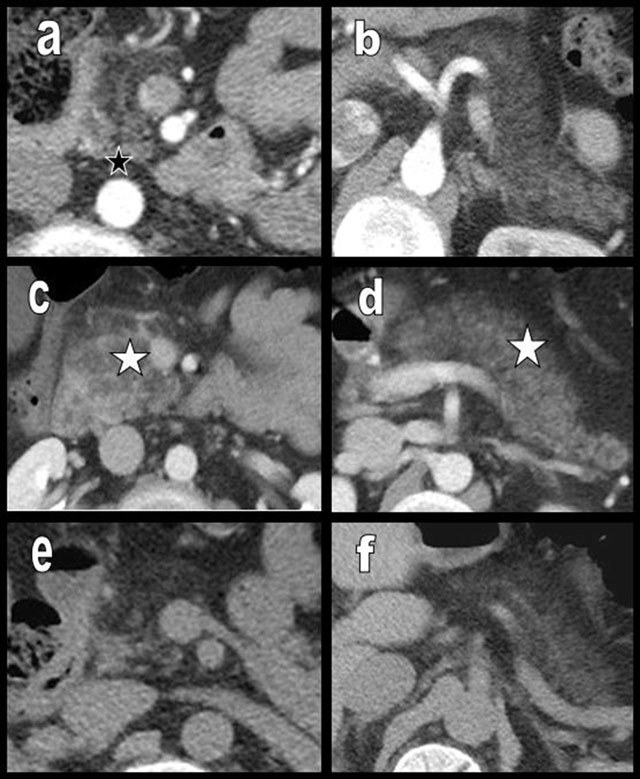
Global FI (type 2b) in a 45-year-old patient (a and b) free from obesity, hypermetabolic state, or diabetes mellitus. The entire pancreas is affected by FI except a small portion of the PPH (black star on a). Three years later, the patient presents with diffuse edematous pancreatitis (c and d) of unknown origin. The gland is swollen with hazy contours, and the density has increased due to edema (white star on c and d). The cephalic portion is particularly affected (c). Four months after the episode of pancreatitis, the morphology of the lipomatous pancreas has returned to the initial state (e and f).

**Figure 21:**
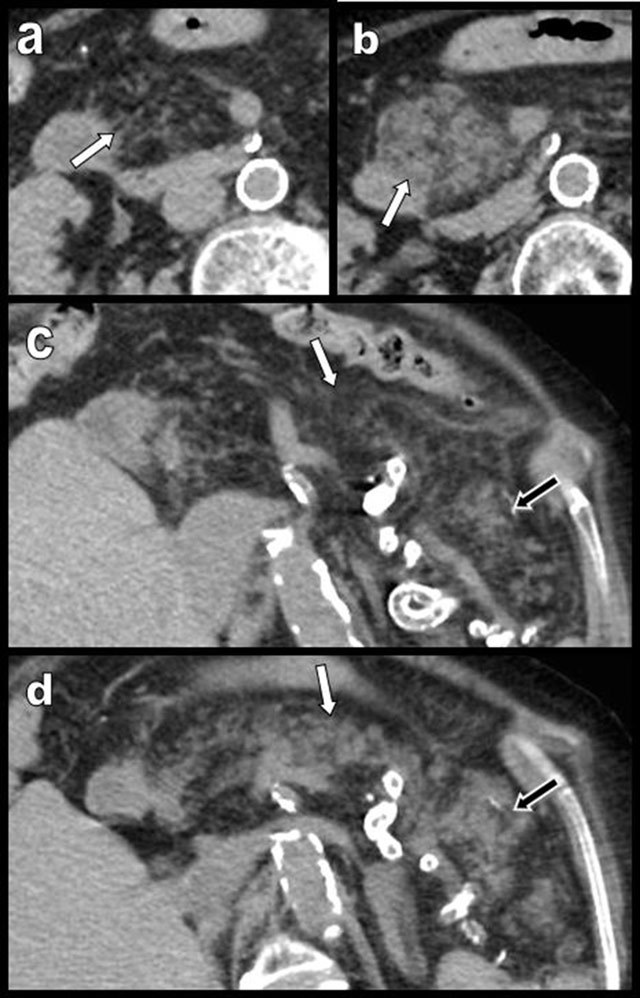
A 60-year-old woman presented with clinical and biological acute episode of pancreatitis. Unenhanced abdominal CT showed a moderately lipomatous but nearly normal cephalic (b) and corporeo caudal (d) pancreatic gland. However, on a previous CT found in the database, the pancreatic gland appeared extremely lipomatous (type 2b) (a and c). Due to edema, acute pancreatitis was responsible of the very unusual “reappearance” of the gland. Acute pancreatitis on a very lipomatous pancreas may mimic a normal pancreas.

FI of the pancreas may also be complicated by acute pancreatitis (Figure 20). In other words, FI of the pancreas does not protect the organ from an episode of pancreatitis. Nevertheless, in these cases, the classical imaging features of inflammation may be altered by the FI background and diagnostic confusion may result. Focal acute or subacute pancreatitis may mimic a tumoral mass due to the focal increase of density of a previously lipomatous portion (Figure 14). In these cases, follow-up is necessary. In the same way, diffuse pancreatitis may mimic a nearly normal pancreas in the case of massive preexisting FI, especially if no previous imaging of the pancreas is available for comparison (Figure 21).

**Figure 22:**
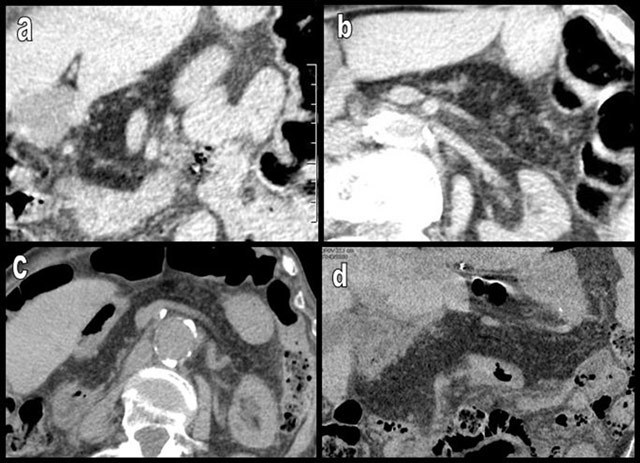
Two cases (a and b, case 1; c and d, case 2) of massive diffuse (type 2b) pancreatic FI in old patients. These patients were very slim with a very reduced quantity of intra-abdominal fat sharpIy contrasting with the persistence of a normal volume of the lipomatous pancreatic bed. The mean density of the pancreatic fatty bed also appeared drastically lower than that of the intra-abdominal fat. The discrepancy was about 25 to 35 U.H. This aspect was probably due to real histologic fatty repIacement (instead of a fatty infiltration). The conservation of the volume of the pancreatic bed could also be explained by the fact that this fatty replacement is more refractory to weight loss.

In our practice, we have experienced rare situations of massive LI of the pancreas – especially in the elderly – in which the density of the fat was extremely low when compared with the rest of the intraabdominal fat (Figure 22). These patients had a reduced amount of intraabdominal fat, probably due to chronic weight loss. Nevertheless, the volume of the pancreatic bed remained normal. The reason for this very low hypodensity is unknown, but we speculate this aspect represents real histologic fatty replacement rather than fatty infiltration. The conservation of the volume of the gland could also be explained by the fact that this fatty replacement is more refractory to weight loss.

## Differential Diagnosis

### Pancreatic Agenesis

**Figure 23:**
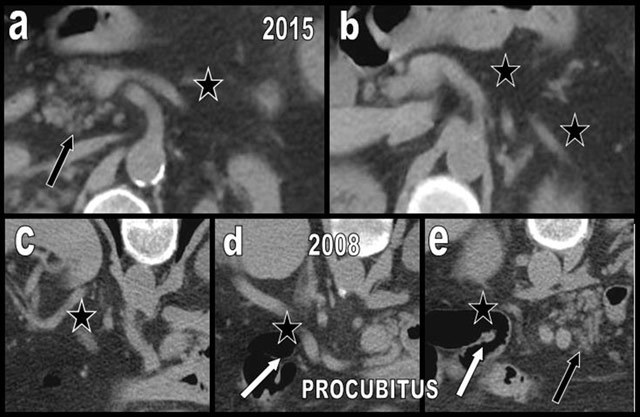
Agenesis of corporeal pancreas (a–e). Just the lipomatous head is present (black arrow on a). The ductal system of the corporeal caudal pancreas is absent (black stars), and the pancreatic bed is empty, allowing bulging of neighboring structures – spIenic vessels and jejuna loops – within the pancreatic bed. Bulging of neighbouring structures within the pancreatic bed is also clearly illustrated (white arrows) on these prone views (c–e) obtained during virtual colonoscopy 7 years before.

FI of the dorsal caudal pancreas must be distinguished from dorsal pancreatic agenesis (PA). In PA (Figure 23), the ductal structure, the islets of Langerhans, and the pancreatic vasculature are absent. On the contrary, these structures are generally preserved in corporeal caudal FI, but exceptions have been reported [22]. Another crucial difference is the conservation of the normal distance between the splenic vein and the gastrointestinal structures in FI. In PA, the potential space of the pancreas – the bed of the pancreas – is filled by gastrointestinal structures (essentially the stomach and intestinal loops).

### Lipomatous Pseudohypertrophy of the Pancreas

**Figure 24:**
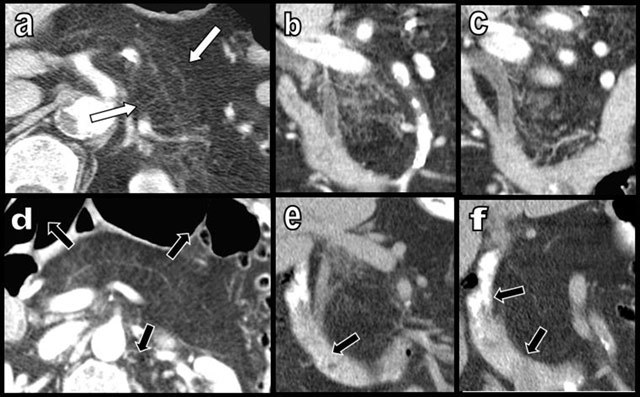
Two typical cases of “lipomatous pseudohypertrophy” of the pancreas. Images a (axial view), b, and c (coronal cephalic views) in an 80-year-old woman with biological pancreatic exocrine insufficiency. FI is maximal, and only the pancreatic ductal network remains visible. The volume of the pancreatic bed has sharply increased (white arrows). Images d (axial view), e, and f (coronal cephalic views) in an 84-year-old obese woman with diabetes and mild biological pancreatic exocrine insufficiency. Pseudohypertrophy of the lipomatous pancreas is marked, with diffuse mass effect on the neighbouring organs (black arrows). The ductal network itself has a ghostly appearance.

Lipomatous pseudohypertrophy (LPH) of the pancreas is a particular situation of FI that has probably been considered in a contestable way as a rare, specific, and distinct entity. This situation of disproportionate replacement of the entire pancreas with increasing amounts of adipose tissue and the subsequent enlargement of the entire gland was first described by Hantelmann in 1931; the disease was named later LPH [23,24] (Figure 24).

The disease is considered very rare, and the specific etiology remains unknown [25]. Associations with rare childhood syndromes such as the Shwachman-Diamon, Bannayan, or Johansson-Blizzard have been reported [3]. Various possible causes have been postulated, ranging from a congenital anomaly to an acquired condition due to injury by infective or toxic agents [3] or caused by chronic obstruction of the pancreatic ducts, causing atrophy and subsequent fatty replacement [26]. This last hypothesis suffers from the fact that the amount of fat is really disproportionate and by the demonstration of a normal pancreatic duct in several articles [23]. Moreover, the residual islets of pancreatic tissue appear rather preserved or at least uninjured [3]. Relations with chronic hepatitis B and other chronic, advanced hepatic lesions have also been reported [3]. This situation has been diagnosed in young patients and in other patients with none of obesity, diabetes mellitus, or pancreatitis. These characteristics probably underscore the benign course of this specific entity that may, however, be associated with considerable pancreatic exocrine dysfunction [23].

## Conclusion

FI is the most common benign condition of the pancreas. The precise etiology remains unknown and may be multifactorial. The diagnosis is classically unambiguously made by ultrasound, CT, and MRI in the vast majority of cases. Uneven FI represents the majority of cases. As a consequence, the imaging features may be extremely varied. “Ductal” and “embryologic” reasons have been proposed to explain these various patterns. The differential diagnosis concerns include agenesis and lipomatous pseudohypertrophy of the pancreas.

## Competing Interests

The author declares that they have no competing interests.
